# Supplementary material for: Function and Regulation of Chloroplast Peroxiredoxin IIE
Source: Antioxidants (Basel). 2021 Jan 21;10(2):152. doi: 10.3390/antiox10020152 (PMC7909837; doi:10.3390/antiox10020152)
Supplement: Supplementary file 1 [file antioxidants-10-00152-s001.zip › antioxidants-1008472-supplementary 3.pdf]

Table S1. Primers used in this study for cloning.

Gene	AGI	Restriction enzyme		Primer Sequence 5′->3′
GRX-S12	AT2G20270	NdeI	Forward	TTTCCAACATATGGGATCGACATTGGAGGAGACTG
		BamHI	Reverse	TTTCAGGATCCCTAGGTCTGACCGTTTTTTCC
SRX	AT1G31170	NdeI	Forward	ACATATGAACGGTTCGCCGCCGGTGAT
		BamHI	Reverse	AAAGGATCCTCAGCGAAGATGATGCCTTA
PRX-IIE	AT3G52960	NdeI	Forward	ATATACATATGGCCTCCATTTCCGTCGG
		BamHI	Reverse	ATATAGGATCCTCAGAGAGCTTTAAGCATATC
PRX-IIE C121S	AT3G52960		Forward	GGCGCATTCACACCAACAAGCTCAC
			Reverse	GTGAGCTTGTTGGTGTGAATG
PRX-IIE C146S	AT3G52960		Forward	AATCGCAAGTATCTCCGTCAAC
			Reverse	GACGGAGATACTTGCGATTACAT
PRX-IIE S82D	AT3G52960		Forward	GGTAGGAGAGAGTGTCGTCTGGGAGCTTGT
			Reverse	ACAAGCTCCCAGACGACACTCTCTCCTACC
PRX-IIE T108E	AT3G52960		Forward	GAACGGCGAATAGGATTTCTTTCTTCCCGGCG
			Reverse	CGCCGGGAAGAAAGAAATCCTATTCGCCGTTC
PRX-IIE T223E	AT3G52960		Forward	CTCAGCACTACTATTTTCAAAAGCACCTCCTTC
			Reverse	GAAGGAGGTGCTTTTGAAAATAGTAGTGCTGAG
PRX-IIE-EYFP	AT3G52960	BamHI	Forward	ATATAGGATCCATGGCGACTTCTCTCTCCGTTTC
		AgeI	Reverse	AAACCGGTGGGAGAGCTTTAAGCATATCCTCAG

Table S2. GSNO-dependent posttranslational modification of PRX-IIE. 10 μM pre-reduced PRX-IIE protein was incubated with indicated concentrations of GSNO for 16 min at RT and analyzed by ESI-MS. Data are means of n= 10 ± SD with the protein of two independent protein purifications.

Treatment/Cys modification	Mass [Da]	Mass difference to reduced protein [Da]
**5 mM DTT** (control)		
-SH	19,438.50 ± 0.38	/
**1 mM GSNO**		
-SNO	19,469.46 ± 0.32	31
-SNO (2x)	19,496.40 ± 0.98	58
-SSG	19,744.42 ± 0.58	306
-SSGNO	19,772.86 ± 1.03	334
**5 mM GSNO**		
-SNO	19,469.37 ± 0.34	31
-SNO (2x)	19,496.77 ± 0.54	58
-SSG	19,744.01 ± 1.05	306
-SSGNO	19,722.80 ± 1,09	334

Table S3. Identified 14-3-3 proteins and their unique peptides.

Protein	AGI	Identified unique peptides	All identified peptides	Unique peptide sequences	Localization	Reference
14-3-3 χ	AT4G09000	11	25	DEFVYMAKLAEQAER<br>DEFVYMAKLAEQAERYEEMVEFMEK<br>DNLTLWTSDMQDDVADDIK<br>DSTLIMQLLRDNLTLWTSDMQDDVADDIK<br>DSTLIMQLLRDNLTLWTSDMQDDVADDIKEAAPAAAKPADEQQS<br>EESRGNDDHVSLIRDYR<br>GNDDHVSLIR<br>GNDDHVSLIRDYR<br>IETELSDICDGILK<br>KDAAEHTLTAYK<br>MATPGASSARDEFVYMAKLAEQAER	Cytosol, nucleus, chloroplast, Golgi, vacuole	[40-44]
14-3-3 ω	AT1G78300	0	8		Cytosol, nucleus	[45]
14-3-3 φ	AT1G78300	4	14	DNLTLWTSDMQDESPEEIK<br>EEFVYLAKLAEQAERYEEMVEFMEK<br>GNDDHVTTIR<br>LAEQAERYEEMVEFMEKVAEAVDK	Cytosol, nucleus, plasma membrane	[41,46,47]
14-3-3 υ	AT5G16050	8	17	ASWRIISSIEQKEDSR<br>DNLTLWTSDLNDEAGDDIKEAPK<br>DSTLIMQLLRDNLTLWTSDLNDEAGDDIK<br>EDSRGNSDHVSIIK<br>ICDGILNLLEAHLIPAASLAESK<br>LGLALNFSVFYYEILNSSDR<br>SAQDIALADLAPTHPIRLGLALNFSVFYYEILNSSDRACSLAK<br>VDEQAQPPPSQ	Chloroplast, cytosol	[48]
14-3-3 λ	AT5G10450	5	7	DQYVVYMAKLAEQAERYEEMVQFMEQLVTGATPAEELTVEER<br>QAFEEAIAELDTLGEESYK<br>QAFEEAIAELDTLGEESYKDSTLIMQLLR<br>YEEMVQFMEQLVTGATPAEELTVEER<br>YMAEFK	Nucleus, cytosol, plasma membrane, chloroplast	[49-51]
14-3-3 ν	AT3G02520	2	12	MSSSREENVYLAK<br>TVDTDELTVEERNLLSVAYK	Chloroplast, Cytosol	[48]

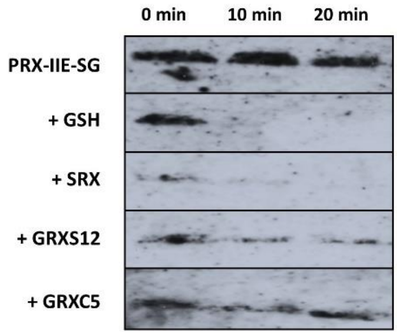

Figure S1. Deglutathionylation of PRX-IIE catalyzed by GSH, SRX, GRX-S12, or GRX-C5. The time course of the deglutathionylation reaction was analyzed by immunodetection with the anti-glutathione antibody. The signal intensity decreased in the presence of GSH and SRX and to a minor extent with GRXS12 as well as with GRXC5, indicating that GSH alone modulates the deglutathionylation of PRX-IIE efficiently.
